# What One Secretary Is Thinking

**Published:** 1917-10-13

**Authors:** 


### WHAT ONE SECRETARY IS THINKING.

**(We publish each paragraph practically as received, withou comment. Contributions to this Column and discussion on the points raised will be welcomed.)**

### "They All Go Too Far."

Two much-respected gentlemen, who are no longer young and who are chairmen of two large charitable institutions, were exchanging personal views at their club. Said one: "My experience is that they all go too far. I've seen four of 'em, and they were all alike. You get a keen fellow and he works hard and you encourage him. Then he begins to take liberties. You find he is competent (even clever), and you make the mistake of leaving too much to him. He begins to think and believe that he is running the hospital. The result is that you have to pull him up and put him in his proper place. Then he becomes saucy. There is a row. He is proud and you are determined. So off he goes." There is a pause. Presently the second much-respected gentleman, who has been reflecting deeply, begins: "Well, you know, it's a bit rough on him. He has to give up a soft job because you spoilt him. You don't suffer. He and the hospital are punished for your slackness. Why did you leave too much to him? Why not have a frank explanation and then shake hands and admit that you were also to blame? Why should one bear all the blame and condemnation? You can't expect a man, or a horse, or a dog, to be blind to the fact when he has a weak master."

